# Outcome prediction with a social cognitive battery: a multicenter longitudinal study

**DOI:** 10.1038/s41537-021-00160-5

**Published:** 2021-05-26

**Authors:** Eric Brunet-Gouet, Capucine Decaix-Tisserand, Mathieu Urbach, Nadine Bazin, Bruno Aouizerate, Lore Brunel, Delphine Capdevielle, Isabelle Chereau, Caroline Dubertret, Julien Dubreucq, Guillaume Fond, Christophe Lançon, Sylvain Leignier, Jasmina Mallet, David Misdrahi, Sylvie Pires, Priscille Schneider, Franck Schurhoff, Hanan Yazbek, Anna Zinetti-Bertschy, Christine Passerieux, Paul Roux

**Affiliations:** 1 FondaMental Foundation, Créteil, France; 2 Department of Adult Psychiatry, Centre Hospitalier de Versailles, Le Chesnay, France; 3 DisAP, DevPsy, CESP, Inserm 1018, University Paris Saclay, INSERM, Villejuif, France; 4 Department of Adult Psychiatry, Charles Perrens Hospital, Bordeaux, France; 5 Laboratory of Nutrition and Integrative Neurobiology (UMR INRA 1286), University of Bordeaux, Bordeaux, France; 6 INSERM U955, Translational Psychiatry Team, Creteil, France; 7 Departement Medico-Universitaire de Psychiatrie et d’Addictologie (DMU ADAPT), Federation Hospitalo-Universitaire de Médecine de Precision (FHU IMPACT), AP-HP, HU Henri Mondor, Creteil, France; 8 University Department of Adult Psychiatry, Hospital La Colombière, CHU Montpellier, Montpellier, France; 9 INSERM, Univ Montpellier, Neuropsychiatry: Epidemiological and Clinical Research, Montpellier, France; 10 University of Montpellier, Montpellier, France; 11 CHU Clermont-Ferrand, Service de Psychiatrie B, Université Clermont Auvergne, Clermont-Ferrand, France; 12 AP-HP; Department of Psychiatry, Louis Mourier Hospital, Colombes, France; 13 Inserm UMR1266, Institute of Psychiatry and Neuroscience of Paris, University Paris Descartes, Paris, France; 14 Sorbonne Paris Cité, Faculté de Médecine, Université Paris Diderot, Paris, France; 15 Psychosocial Rehabilitation Reference Centre, Alpes Isère Hospital, Grenoble, France; 16 La Conception Hospital, AP-HM, Aix-Marseille Univ., School of Medicine—La Timone Medical Campus, EA 3279: CEReSS—Health Service Research and Quality of Life Center, Marseille, France; 17 Ste Marguerite Hospital, AP-HM, Aix-Marseille Univ., School of Medicine—La Timone Medical Campus, EA 3279: CEReSS—Health Service Research and Quality of Life Center, Marseille, France; 18 Department of Psychiatry, University Hospital of Strasbourg, Strasbourg, France; 19 University of Strasbourg, Strasbourg, France; 20 Inserm U1114, Strasbourg, France

**Keywords:** Schizophrenia, Human behaviour

## Abstract

The interest in social cognition in schizophrenia is justified by the relationship between deficits in these skills and negative functional outcomes. Although assessment batteries have already been described, there is no consensus about which measures are useful in predicting patient functioning or quality of life (QoL). We investigated a set of five measures of recognition of facial emotions, theory of mind (ToM), and empathy in a cohort of 143 patients with schizophrenia or schizoaffective disorder at inclusion and, amongst whom 79 were reassessed 1 year later. The distribution was satisfactory for the TREF (Facial Emotion Recognition Task), V-SIR (Versailles-Situational Intention Reading), and QCAE (Questionnaire of Cognitive and Affective Empathy). Internal consistency was satisfactory for the TREF, V-SIR, V-Comics (Versailles Intention Attribution Task), and QCAE. Sensitivity to change was acceptable for the TREF. The TREF and V-SIR showed a cross-sectional relationship with functioning beyond the clinical symptoms of schizophrenia but not beyond neurocognition. Moreover, the TREF and V-SIR at inclusion could not predict functioning one year later, whereas most neurocognitive and clinical dimensions at inclusion could. Finally, only affective QCAE showed a significant cross-sectional, but not longitudinal, association with QoL. In conclusion, the TREF had satisfactory psychometric properties and showed a cross-sectional, but not longitudinal, association with objective outcome measures, thus appearing to be reliable in clinical practice and research. The V-SIR also showed promising psychometric properties, despite a possible weakness to detect change. However, these measures should be interpreted within the context of the good predictive power of the neurocognitive and clinical status on the outcome.

## Introduction

The ongoing interest in the constructs of social cognition in schizophrenia is justified by the relationships between deficits in these skills and a negative functional outcome^1^. Several authors have postulated that social cognition is a distinct construct that acts as a mediator between cognitive functions and functioning, despite its close association with neurocognition^2,3^. In a meta-analysis, Fett et al.^4^ reported that social cognitive deficits in schizophrenia have a greater impact on social functioning than neurocognitive disorders (i.e., memory, attention, executive functioning, and speed of processing). Another meta-analysis showed that social cognition explains a larger portion of the variance in functioning than neurocognition^5^. Because of its importance as a determinant of functional and clinical outcomes, social cognition could therefore be considered as a cognitive domain per se, thus justifying its specific evaluation along with that of neurocognition^6^. Departing from a single domain conceptualization, Green et al.^7^ considered that five constructs characterize social cognition: perception of emotions, attributional style, theory of mind (ToM), social perception, and social knowledge. To date, the use of several tools organized within a battery of tests has been a logical response to the inherent heterogeneity of these constructs and has encouraged psychometric research to measure the advantages and weaknesses of candidate measures.

Within a classical psychometric description, an evaluation tool is expected to meet certain criteria based on its own characteristics: the results showing a normal distribution, not showing a floor or ceiling effect, showing satisfactory internal consistency, and test–retest reliability, and not showing a systematic trend between test and retest, as evaluated over short periods of time (utility as a repeated measure). These various psychometric characteristics have been reported in several studies but have not yet led to a consensual proposal, although it is acknowledged, however, that certain instruments may be considered to be acceptable^8–11^.

Another important characteristic for evaluating the health status over a long period is the sensitivity to change (sometimes called responsiveness). Such sensitivity is defined as the extent to which an evaluation tool can detect a change when it has occurred, irrespective of whether the change is meaningful^12^. It provides certainty that an improvement measured by an instrument is due to a true change and not random error^13^. The sensitivity to change of the various tools used to assess social cognition has not yet been investigated in schizophrenia.

Beyond the requirement of being internally adequate, the value of an assessment tool is also recognized through its properties of concurrent/divergent validity. Based on the principle that cognitive deficits causally precede behavior, social cognitive measures are often assessed for their power to predict outcome measures, such as real-life functioning. In several types of research, criterion validity is described as the relationship between the social cognitive test and an outcome, such as a performance-based assessment^11,14^, or real-life functioning^8,14^.

As mentioned above, social cognition is associated with neurocognition; these two types of performance both partially predicting functioning. Consequently, it is informative to characterize incremental validity as the predictive power of a measure beyond neurocognition. Although most social cognitive measures included in the SCOPE battery exhibit significant cross-sectional correlations with certain outcome measures, only a few (ER-40, Hinting task, and IBT) show additional predictive power beyond neurocognition^11^. Such added value was also found for the DACOBS and TASIT Lies^8^ but was not supported in another study^14^. However, these studies measured the strength of association between social cognition and functioning using either a cross-sectional design or during a short period (no longer than 4 weeks). Arguably, a much more extended period would be required to reveal the action of causal pathways and capture changes related to disease progression and therapeutic interventions.

Considering the incomplete information in the literature about the psychometry of social cognitive measures, the first objective of the present study was to characterize the properties of a comprehensive set of social cognitive measures in individuals with schizophrenia spectrum disorders. Besides distribution-based psychometric properties, we investigate sensitivity to change through a 1-year reassessment of patients. The second objective was to add knowledge on these measures’ predictive capacity of the outcome. For the first time in our knowledge, functioning and quality of life (QoL) will be considered cross-sectionally and longitudinally over one year. Last, we bring thorough insights into each measure’s predictive power beyond neurocognition thanks to a complete neuropsychological assessment.

## Results

The study included 143 patients with schizophrenia (*n* = 103) or schizoaffective disorder (*n* = 40), who were included between April 2013 and June 2017. Among them, 79 participants were reassessed one year later, representing a cohort attrition rate of 55%.

### Description of the population

The population was predominantly male, with an average educational level between high school and university degrees (see Table 1). The average total PANSS score indicated mild symptoms, and the severity of the disorder (CGI) was moderate. No differences were found between completers and non-completers for the clinical, neuropsychological, and social-cognitive profiles at inclusion (see Supplementary Table 1), thus suggesting a lack of attrition bias in the sample.

**Table 1:** Baseline clinical characteristics of the participants.

Characteristics	*n*	%
Males	111	77.6
Schizophrenia	103	72.7
Schizo-affective disorder	40	27.3
	Mean	SD
Age (years)	31.4	8.2
Educational level (years)	12.6	2.4
Premorbid IQ	104	8
Age at onset of illness (years)	21.4	5.6
Duration of untreated psychosis (years)	1.5	3
Total duration of hospitalization (months)	5.9	7.2
PANSS total score	57.6	18.9
Antipsychotics in chlorpromazine equivalents (mg/24 h)	460.7	386.4
CGI severity	4.1	1.4
PSP	52.6	17.5
S-QoL	50.1	19.8

*PANSS* positive and negative syndrome scale, *CGI severity* clinical global impression-severity scale, *PSP* personal and social performance scale, *S-QoL* schizophrenia quality of life questionnaire.

### Neurocognitive status

Among the participants, 63.6% were evaluated with the WAIS-III and 36.4% with the WAIS-IV. The neurocognitive performance of the sample is described in Supplementary Table 2. The most strongly affected cognitive dimensions were processing speed (−0.8 ± 0.7 SD), memory (−0.9 ± 0.9 SD), and executive functions (−1 ± 1.5 SD). Attention (−0.4 ± 0.5 SD), working memory (−0.5 ± 0.7 SD), and reasoning (−0.4 ± 0.9 SD) were strongly less affected.

### Psychometric analysis of social cognitive measures

The psychometric data for the EVACO social cognition measures are summarized in Table 2. The distribution was satisfactory for three measures: test de reconnaissance des émotions faciales—facial emotions recognition task (TREF), versailles-situational intention reading (V-SIR), and questionnaire of cognitive and affective empathy (QCAE). The two measures of the Social cognition, Perception, eXecutive functions—goal & belief attribution sensitivities (SPEX-GBA) test showed a floor effect. Conversely, the V-Comics suffered from a ceiling effect. All outlier data of the EVACO battery showed extremely low performance. The two versions of the V-Comics had the greatest number of outliers (7 for versions 1 and 2). The V-SIR task, version 1 of the SPEX-GA test, and cognitive empathy had a small number of outliers (2, 1, and 1, respectively), whereas the other tasks had none.

**Table 2:** Psychometric characteristics of social cognition measures.

Dimension	Instrument	*N*	Mean	SD	Floor (%)	Ceil (%)	Outliers	Symmetry coefficient
Facial emotion recognition	TREF	114	35.8	5.8	0	0	0	−0.55
Theory of mind	SPEX- BA V1	51	1.3	1.3	25.5	11.8	0	−0.58
	SPEX- BA V2	51	1.1	1.2	31.4	11.8	0	0.19
	SPEX-GA V1	51	1.4	1.3	21.6	19.6	1 (lower)	−0.59
	SPEX-GA V2	51	1.3	1.1	25.5	11.8	0	0.08
	V-SIR	140	17.4	5.9	0	0	2 (higher*)	0.82
	V-Comics V1	72	11.6	2.2	2.8	19.4	7 (lower)	−0.95
	V-Comics V2	71	12.52	1.73	2.8	42.3	7 (lower)	−0.97
Self-reported empathy	QCAE affective	137	2.29	0.39	0	0	0	−0.27
	QCAE cognitive	137	2.29	0.46	0.7	0	1	−0.20

*TREF* test de reconnaissance des émotions faciales—facial emotions recognition task, *SPEX-BA & GA* social cognition, perception, eXecutive functions—belief and goal attribution sensitivities, *V-SIR* versailles-situational intention reading, *V-Comics* versailles intention attribution task, *QCAE* questionnaire of cognitive and affective empathy, *N* number of data points, *SD* standard deviation.

*For V-SIR, higher scores reveal lower performance.

Internal consistency was satisfactory for most EVACO tasks: the TREF with *α* = 0.76 [0.70–0.82], the V-SIR with *α* = 0.74 [0.68–0.72], the V-Comics with *α* = 0.74 [0.66–0.83] for version 1 and *α* = 0.83 [0.77–0.88] for version 2, and the QCAE cognitive empathy with *α* = 0.87 [0.83–0.90]. Internal consistency was insufficient for the SPEX-GA test, with *α* = 0.61 [0.48–0.75] for version 1 and *α* = 0.31 [0.08–0.55] for version 2, the SPEX-BA test, with *α* = 0.52 [0.35–0.68] for version 1 and *α* = 0.38 [0.17–0.60] for version 2, and QCAE affective empathy, with *α* = 0.62 [0.53–0.71].

### Sensitivity to change

First, we have tested for differences between inclusion and follow-up in clinical and functional outcomes for participants who completed both the inclusion and follow-up. Patients showed an improvement in outcomes related to illness, with a decrease in their symptoms for all PANSS dimensions (paired *t* tests, *p* < 0.05) except excitation (paired *t* test, *p* = 0.17). The severity of their illness decreased (paired *t* test, *p* < 0.001), while functioning and QoL improved (paired *t* test, *p* = 0.001; Supplementary Table 3). This table also reported the correlation of clinical and functional outcomes between inclusion and follow-up for participants who completed both evaluations. The sensitivity to change was medium for the TREF (standardized response mean (SRM) = 0.43), small for the V-SIR and QCAE (see Table 3), and not investigated for the remaining tools included in the EVACO battery. The sensitivity to change for the neurocognitive measures are reported in Supplementary Table 4. Only the global performance index of TAP-flexibility showed a large sensitivity to change (SRM = 0.59). The TMT, CPT-IP, TAP-Alterness (SD of reaction times with alert), TAP-flexibility (number of errors), and TAP-divided attention (number of errors) all showed moderate sensitivity to change. The remaining neurocognitive measures that were reassessed at 1 year showed only small sensitivities to change.

**Table 3:** Social cognitive performances at inclusion and follow-up and standardized responses means in individuals who completed both visits.

Variable	Inclusion		Follow-up		SRM
	Mean	SD	Mean	SD	
TREF	35.4	6.4	35	8.1	0.43
V-SIR	17.5	6	17.5	6.6	0.01
QCAE affective	2.3	0.4	2.3	0.4	−0.14
QCAE cognitive	2.3	0.3	2.3	0.4	−0.08

*SRM* standardized response mean, *TREF* test de reconnaissance des émotions faciales—facial emotion recognition task, *V-SIR* versailles-situational intention reading, *QCAE* questionnaire of cognitive and affective empathy.

### Cross-sectional relationships between social cognitive measures and outcomes

**Fig. 1:**
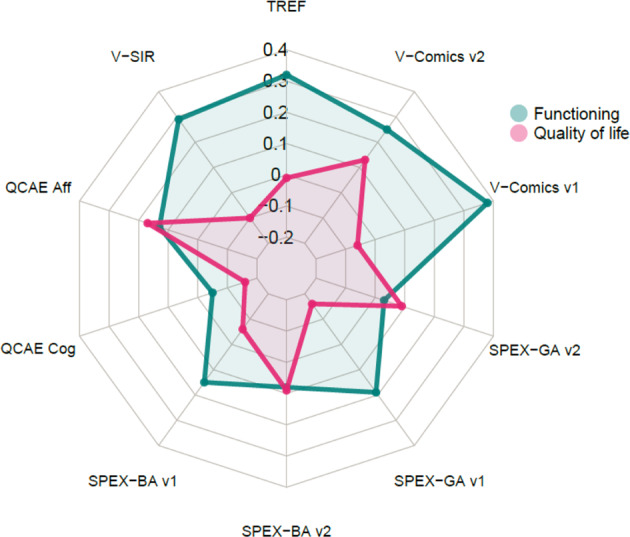
ß standardized coefficients of simple cross-sectional regressions of functioning (PSP) and quality of life (S-QoL) on various social cognition variables at inclusion. TREF, Test de Reconnaissance des Émotions Faciales—Facial Emotion Recognition Test, V-Comics: Versailles Intention Attribution Task, SPEX-GA & BA: Social cognition, Perception, eXecutive functions—Belief & Goal Attribution sensitivities, QCAE Cog: Questionnaire of Cognitive and Affective, QCAE aff: Questionnaire of Cognitive and Affective Empathy—Affective empathy subscore Empathy—Cognitive empathy subscore, V-SIR: Versailles Situational Intention Reading. The signs of ß were reversed for V-SIR.

Cross-sectional simple regressions for functioning for the various social cognitive variables showed significant results for the TREF (*ß* = 0.32, *t*(119.8) = 3.7, *p* < 0.001), V-SIR (after sign reversion: *ß* = 0.29, *t*(133.2) = 3.5, *p* = 0.001), and V-Comics version 1 (*ß* = 0.38, *t*(61.7) = 3.6, *p* = 0.001). Better performance in social cognition was associated with better functioning (see Fig. 1, Supplementary Table 5). Significant associations with functioning were also found for all neurocognitive variables (0.2 ≤ *ß* ≤ 0.34, Supplementary Table 6) and clinical variables (−0.41 ≤ *ß* ≤ −0.25, Supplementary Table 7).

Hence, we further investigated the relationships between social cognitive variables and functioning, successively taking into account clinical variables and neurocognition. First, the TREF, V-SIR, and V-Comics remained significant predictors of functioning, regardless of the clinical dimension (positive, negative, disorganization, depression, or excitation) introduced into the model as a covariate (Supplementary Table 8). Second, the association of functioning with the TREF remained significant, regardless of the neurocognitive dimension included as a covariate (Supplementary Table 9).

Third, the association of functioning with the V-SIR, which was significant in the simple analyses, became nonsignificant (*p* = 0.125) when reasoning was included as a covariate and marginally significant (*p* = 0.065) when executive functions were included. Finally, the association of functioning with the V-Comics version 1 became marginally significant (*p* = 0.082) when reasoning was included as a covariate.

Among social-cognitive predictors, only the affective score of the QCAE exhibited a significant relationship with QoL (*ß* = 0.17, *t*(134.3) = 2, *p* = 0.044), the cognitive score being marginally negatively significantly related (*ß* = −0.16, *t*(131.7) = −1.8, *p* = 0.07, see Fig. 1, Supplementary Table 10). No cognitive variable showed a significant relationship with QoL (Supplementary Table 11). There were significant negative relationships of QoL with positive, negative, and depression PANSS scores, but not with disorganization nor excitation (Supplementary Table 12).

We explored the relationships of affective QCAE with QoL, introducing clinical covariates to find potential confounders (Supplementary Table 13). The link between affective QCAE and QoL became non-significant when depression was introduced as a covariate. This link became marginally significant with positive and negative dimensions as covariates and remained significant with disorganization and excitation as covariates.

### Predicted 1-year outcome

**Fig. 2:**
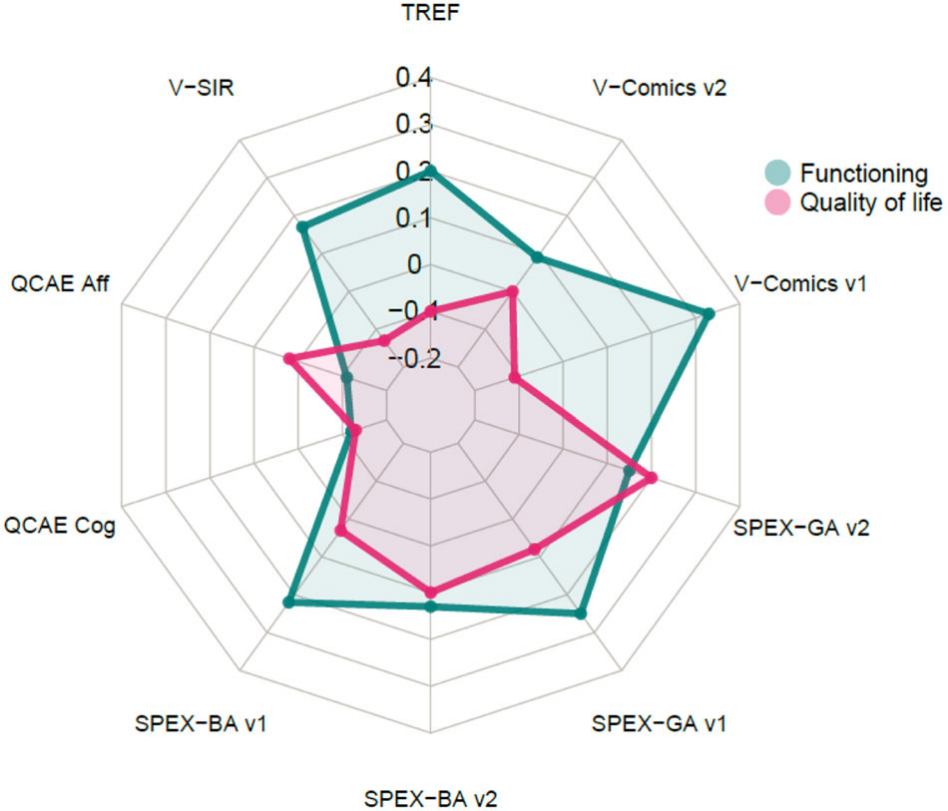
ß standardized coefficients of simple regressions of functioning (PSP) and quality of life (S-QoL) at follow-up on various social cognition measures at inclusion. Legends: please see Fig. 1.

Simple longitudinal regressions of functioning at follow-up on social cognition at inclusion showed significant results only for the V-Comics version 1 (*ß* = 0.33, *t*(40.3) = 2.6, *p* = 0.013), with good skills in ToM at inclusion related to better functioning at one year (see Fig. 2, Supplementary Table 14). In contrast, regression of functioning at follow-up on neurocognitive variables at inclusion gave more consistent results, with significant positive associations for all neurocognitive dimensions, except memory (Supplementary Table 15). Similarly, most baseline clinical dimensions (PANSS positive, disorganization, excitation, and depression but not negative dimension) showed significant negative relationships with functioning at follow-up (Supplementary Table 16). Functioning at follow-up was also predicted by functioning and QoL at inclusion (Supplementary Table 16).

The prediction of functioning at follow-up by social cognition at inclusion beyond neurocognition at inclusion is summarized in Supplementary Table 17. The prediction of functioning at follow-up by the V-Comics version 1 at inclusion became insignificant when almost any neurocognitive domain was included as a covariate (processing speed, working memory, reasoning, and executive functions), except for memory, for which the relationship became marginally significant. Finally, the prediction of functioning at follow-up by version 1 of the V-Comics at inclusion became marginally significant when the positive disorganization and excitation dimensions were included as covariates. The association remained significant when negative symptoms, depression, and QoL were included as covariates. The association became nonsignificant when functioning at inclusion was introduced as a covariate (Supplementary Table 18).

There was no relationship between social cognition variables at inclusion and QoL at 1 year (see Fig. 2, Supplementary Table 19). The same negative findings were found for neurocognitive variables (Supplementary Table 20).

## Discussion

The objective of this study was to characterize the psychometric properties of a set of social cognitive measurements, administered as a battery to a population of individuals with schizophrenia spectrum disorders in a longitudinal design and to assess its relevance to predict outcome measures, such as functioning and QoL.

The psychometric characteristics of EVACO instruments are summarized in Table 4. The distribution analysis suggests that the TREF, V-SIR, and QCAE allow good discrimination between individuals, with few outliers. The floor effect for the SPeX-GBA test suggests that the task was too difficult, whereas the V-Comics ceiling effect indicates that the task was too easy, in agreement with the results of a previous study^9^. The V-Comics also suffered from a large number of extremely low performances, which may have been facilitated by its ceiling effect. The results showed good internal consistency for all EVACO tasks, except the SPEX-GBA test and affective subscale of the QCAE.

**Table 4:** Synthesis of the psychometric properties of each tool used in the EVACO battery.

Instrument	Distribution	Floor	Ceil	Internal consistency	Sensitivity to change	Cross-sectional link with outcome	Longitudinal link with outcome
SPEX- BA V1	NA	NA	A	NA	U	NA	NA
SPEX- BA V2	NA	NA	A	NA	U	NA	NA
SPEX- GA V1	NA	NA	NA	NA	U	NA	NA
SPEX- GA V2	NA	NA	A	NA	U	NA	NA
TREF	A	A	A	A	A	A^a,b^	NA
V-SIR	A	A	A	A	NA	A^b^	NA
V-Comics V1	NA	A	NA	A	U	A^b^	A
V-Comics V2	NA	A	NA	A	U	NA	NA
QCAE affective	A	A	A	NA	NA	A^c^	NA
QCAE cognitive	A	A	A	A	NA	NA	NA

*A* acceptable, *NA* not acceptable, *U* unknown,

^a^Significant relationship with quality of life.

^b^Significant relationship beyond neurocognition.

^c^Significant relationship beyond symptoms.

*SPEX-BA & GA* social cognition, perception, executive functions—belief & goal attribution sensitivities, *TREF* test de reconnaissance des émotions faciales—facial emotions recognition task, *V-SIR* versailles-situational intention reading, *V-Comics* versailles intention attribution task, *QCAE* questionnaire of cognitive and affective empathy.

Among the tools we investigated in the EVACO battery, only the TREF showed sufficient sensitivity to change. Several confounding factors may explain the low sensitivity to change found for the V-SIR and QCAE rather than psychometric flaws in these tools. The first factor could be a lack of change in ToM and empathy in the recruited sample. However, this explanation seems unlikely, as schizophrenia symptoms and functioning improved during the follow-up in our study. Indeed, better ToM was previously shown to be associated with lower symptoms and better functioning^15,16^. Similar results were also found for empathy^17–19^. The second factor could be the considerable heterogeneity in the changes of the scores, which may have led to a small SRM and an underestimation of sensitivity to change. Impairment of social cognition is heterogeneous in schizophrenia^20^, which may lead to its heterogeneous course. Further studies investigating sensitivity to change in social cognitive measures should attempt to recruit homogenous samples according to the individual magnitude of social cognition change. Third, the poor sensitivity to change found for the V-SIR and QCAE may be explained by low test-retest reliability, which was not investigated in this study. Reliability is closely associated with sensitivity to change, as tools that are prone to higher variability between assessments may detect less change than those with lower between-assessment variability. Finally, practice effects may interfere with the ability to measure changes in cognition, at least in designs based on several administrations of cognitive tests^21,22^. However, the intervals between evaluations in these previous studies were much shorter than the 1-year follow-up used in this study. This study confirms a previous report that patients followed in the FACE-SZ cohort showed a clinical (positive and negative symptoms, depression, insight, and suicidality) and functional improvement in the first year of follow-up^23^. Increased access to psychosocial rehabilitation interventions such as psychoeducation and cognitive remediation has been associated with an improvement in positive and negative symptoms, neurocognition and functioning in this cohort^24^. Another source of improvement for patients followed in the FondaMental Advanced Centres of Expertize may also be the suggestion of personalized medication adjustment in order to follow as closely up-to-date international recommendations.

The recognition of emotions, assessed using the TREF, was positively associated with functioning beyond neurocognition and symptoms. This result is consistent with previous reports that showed associations between the recognition of emotions, as measured by the ER-40 and BLERT tasks, and functioning beyond neurocognition^8,11,25^.

Among the three tools that evaluate ToM, the V-SIR and V-Comics version 1 showed cross-sectional relationships with functioning beyond symptoms of schizophrenia but not beyond reasoning. Our results are consistent with a meta-analysis that estimated the correlation between ToM and reasoning abilities to be 0.30, corresponding to approximately 10% of common variance^26^. Moreover, another meta-analysis showed reasoning abilities and functioning to exhibit correlation coefficients ranging from 0.08 for community functioning to 0.3 for social problem solving^5^.

The SCOPE study also found an association between ToM (measured by the Hinting Task and RMET) and functioning, but only the Hinting Task showed an association with functioning beyond neurocognition^11^. Davidson et al.^8^ included the V-Comics in their battery and, contrary to our study, did not report such an association with functioning. However, they did not split the task into two versions, and the association that we found was significant for only one.

On the other hand, the SPeX-GBA test, of which the psychometric properties are weaker than those of the other tasks, did not show any association with functioning. Therefore, it does not appear adequate to be included in a social cognition battery. Finally, as assessed by the QCAE, empathetic dispositions did not show a significant association with functioning, contrary to the results reported for the SCAF battery^14^. However, the measurement of empathy in that battery was made using a very different paradigm, closer to a ToM task.

Overall, almost all neurocognitive and clinical variables were associated with functioning in our study. The most predictive neurocognitive dimensions of functioning were, in decreasing order: executive function, reasoning, processing speed, attention, and working memory. Our results suggest that impairments in neurocognition and social cognition share a common variance in their association with functioning. Nevertheless, specific social cognitive measures provide additional information on functioning determinants when added to the neurocognitive and clinical status.

The TREF predicted functioning at one year but not beyond neurocognition. Our results suggest that functioning at follow-up is weakly predicted by social-cognitive measures at inclusion, with only one measure among 10 (V-Comics version 1) being associated with functioning. In contrast, functioning at follow-up was more consistently predicted by neurocognition and symptoms of schizophrenia at inclusion. Moreover, the predictive power of the V-Comics version 1 at baseline on functioning one year later was mainly explained by neurocognition and functioning at baseline. In particular, reasoning, executive functions, and working memory appear to explain the longitudinal link between social cognition and functioning. This may be related to the results of Hoe et al. They used latent difference analysis, and their final multivariate model suggested that baseline neurocognition predicts changes in social cognition at one year, as well as changes in psychosocial functioning^27^. In contrast, baseline social cognition did not predict functioning one year later. These results suggest that the upward causal influence of social cognition in predicting functional outcome fully overlaps with neurocognition.

In the present study, the patients’ QoL was not predicted by any social cognitive or neurocognitive measures, except for self-reported affective empathy (QCAE). The literature provides conflicting results concerning the impact of cognitive processes and empathic dispositions on QoL. Several authors found a positive association between cognitive performance and QoL^28–31^, whereas others found a negative^32–34^ or non-significant association^35,36^. The negative results found for the V-SIR are also consistent with those of a previous study using the same schizophrenia quality-of-life questionnaire (S-QoL) scale^37^. Since the measures have in common the fact that they are self-assessments, an explanatory hypothesis for their common variance could be an undifferentiated effect of this modality.

The following limitation in this study has to be considered. The sample size might have lacked some power, particularly for tools that were divided into two versions (SPEX and V-Comics). Moreover, some instruments, SPEX and TREF, had a higher proportion of missing data than others due to a lack of instrument availability in some centers when the inclusions began. Further study should gather together the instruments selected by the different social-cognitive batteries described in the literature and assess which minimal tool combination captures the optimal amount of social cognition variance using step-wise regression analysis. This study suffers from a 12-month attrition rate of approximately 46%, raising the question of possible bias in the results. We have no information on the reasons for this. However, comparisons of patients’ clinical, neurocognitive, and social-cognitive characteristics according to whether or not they completed the second assessment did not show any difference that would bias the results. Moreover, lower power cannot explain the lower social-cognitive instruments’ ability to predict functioning longitudinally compared to transversal associations. As longitudinal regressions analyses were run after estimating the missing data by multiple imputations, the sample sizes were identical in transversal and longitudinal regression analyses.

To conclude, our results highlight the satisfactory psychometric properties of one measurement from the EVACO battery, the TREF, which showed cross-sectional, but not longitudinal, associations with objective outcome measures beyond symptoms and neurocognition. The TREF thus appears to be reliable for use in clinical practice and research. The V-SIR also shows promising psychometric properties, despite possible weaknesses in the associations with functioning beyond neurocognition and detecting change. The association between social cognition and functioning appears largely explained by specific neurocognitive dimensions, mainly executive function and reasoning, but not clinical symptoms. In contrast, the results of social cognition tests appear to not correlate with QoL, which instead may be associated with self-reported empathetic skills. The evaluation of this construct may be particularly relevant for clinical or research purposes when the outcome of interest depends on the patient’s subjective complaints and not solely on objective capacity.

## Methods

### Procedure

In this multicenter longitudinal study (NCT02901015, previously registered at ClinicalTrial under number NCT02901015), patients were recruited from seven Centers of Expertize for schizophrenia (Clermont-Ferrand, Créteil, Grenoble, Marseille, Montpellier, Strasbourg, and Versailles), making up a network established by the FondaMental Foundation. Patients were referred by their general practitioners or psychiatrists. The local medical ethics committee (*Comité de Protection des Personnes Ile-de-France XI*) approved the study (2012-A00387-36). Each participant gave written informed consent before inclusion and received an indemnity.

### Participants

The primary criterion for inclusion was the presence of schizophrenia or schizoaffective disorder for which the diagnosis was based on a structured clinical interview according to the DSM4-TR (Diagnostic and Statistical Manual of Mental Disorder. 4th edition, revised) criteria^38^. Senior psychiatrists or psychologists conducted the clinical interviews focused on schizophrenia. The patients were between 15 and 65 years of age. The exclusion criteria were current hospitalization in psychiatry or a change in treatment within the last four weeks, substance dependence at the time of assessment (except tobacco), electroconvulsive therapy (for less than 6 months), neurological lesions or pathology that may have significantly influenced cognitive abilities, and significant sensory impairment. Subjects who were unable to attend the various assessments or planned to change their medical follow-up within the following year were also excluded. Participants were assessed at inclusion and one year later.

### Clinical assessment

All the tools and questionnaires were administered by trained clinicians from the different centers. The same clinicians did the evaluations at baseline and follow-up for 96.2% of patients. At least one meeting was organized per year in order to harmonize interrater reliability in clinical assessment between the different centers involved in the recruitment network.

The severity of schizophrenic symptoms was assessed using the Positive and Negative Syndrome Scale (PANSS)^39^. Five PANSS sub-scores were calculated: positive, negative, disorganization, arousal, and depressive symptoms^40^. The PANSS has good internal consistency (Cronbach’s α - CA: 0.73–0.83)^39^ and acceptable interrater reliability (intraclass correlation coefficient—ICC: 0.56–0.80)^41^. The Clinical Global Impression-Severity Scale (CGI-S) assessed the severity of the schizophrenic spectrum disorder^42^.

### Assessment of social cognition

Social cognition was evaluated with a battery (EVACO) of tests and a self-evaluation administered by trained neuropsychologists^43^. The authors selected the tools by considering each paradigm’s characteristics in terms of the tested psychological construct to be the most exhaustive as possible. We included tests that assessed several mental state categories (attribution of intentions, beliefs, and affective and emotional states to others), several attributional stances (3rd person and self-assessment), and several stimuli modalities (static and animated visuals, verbal, and nonverbal content).

To assess facial emotion recognition, the TREF^44^ consists of 54 color photographs of actors’ faces presented on a computer screen. Each picture is displayed for 10 s and depicts an emotion with nine degrees of expressive intensity, obtained by image morphing transformation. The participant must then choose from among six emotions (fear, sadness, contempt, anger, joy, or disgust) that best represent what the actor may be feeling. We used the average accuracy overall conditions.

Theory-of-mind was assessed with V-Comics (Versailles Intention Attribution Task)^45^ that is composed of short comic strips in which the person has to find the most logical ending by choosing among three propositions (one right answer, two wrong answers). This task is composed of three conditions: attribution of an intention to others and understanding physical causality, with or without characters. This task was divided into two versions of equal length (by matching the difficulty of the two versions based on previous results) to produce two alternative versions for test-retest intervention protocols and avoid a learning effect. Each of the two versions has 20 items for the physical logic control condition and 14 items for the intention attribution experimental condition. We used the average accuracy in the intention attribution experimental condition.

The V-SIR^46^ task consists of eight short videos that elicit theory-of-mind judgments. The participant has to judge the degree of probability of four propositions explaining one of the characters’ intentions. This judgment is made on a four-level Likert scale. The score used is the average distance between the participant’s responses and those of a group of control subjects described in a previous study^46^. Higher scores represent lower ToM performance.

The SPEX-GBA theory-of-mind test consists of eight scenarios in the form of silent animations^47^. They are divided among five conditions to evaluate the ability of the test subject to attribute physical causes to an event and the goals and beliefs of a character. As for V-COMICS, this task was divided into two versions of equal length. Each version has 20 items (12 to assess the attribution of goals and beliefs to a character, 8 to assess the attribution of physical causality). In the analyses, only the 12 items assessing the attribution of goals and beliefs were considered. The sensitivity of goal and belief attribution was calculated from these 12 items as described in Supplementary Information 1.

Last, the QCAE^48^ is a self-administered questionnaire consisting of 31 questions that assess empathic dispositions in their cognitive and affective dimensions on a four-level Lickert agreement scale. The average score across all items for the affective and cognitive subscales was used as defined by the authors.

### Assessment of neurocognition

Six domains of neurocognition were evaluated at inclusion (Supplementary Information 2): processing speed, attention/vigilance, working memory, memory, reasoning, and executive functions. For each domain, higher scores reflected better performance after normalization.

### Assessment of outcome

Based on a clinical interview, social functioning was assessed using the personal and social performance scale (PSP)^49^, which provides an assessment of four domains (usual social activities, social and work relationships, self-maintenance, and disruptive and aggressive behaviors). Scores range from 0 to 100, higher scores reflecting better functioning. PSP score is computed from graded severity levels in each domain, all of which being combined into a broad score and then adjusted thanks to the overall opinion of the rater on other domains of social function. The scale has shown acceptable internal consistency (CA, 0.76)^50^ and excellent interrater reliability (ICC, 0.97)^51^.

QoL was assessed using the S-QoL 18^52^, a self-assessment scale with eight factors: psychological well-being, physical well-being, self-esteem, family relationships, relationships with friends, resilience, autonomy, and emotional life. Higher scores reflect a better QoL. Cronbach alpha coefficient is satisfactory 0.72–0.9.

### Statistics

The distributions of each baseline measure of social cognition were tested for normality, the presence of outliers, ceiling or floor effects, and internal consistency. Sensitivity to change between baseline and follow-up was also assessed with the SRM. This characteristic was computed only for participants who completed the inclusion and follow-up and not calculated for tests administered in two different versions at inclusion and follow-up.

The relationships between social cognition variables, functioning, and QoL were explored first at the inclusion visit, and then longitudinally between inclusion and follow-up, after estimating the missing data by multiple imputations (50 imputations, Markoff chain equations) using the MICE package in R^53^.

Simple cross-sectional relationships between social cognition and outcome measures (i.e., functioning, PSP, QoL, and S-QoL) at inclusion were explored using successive univariate linear regressions. Multiple variable linear regression analyses were conducted to determine whether the previously identified links could resist the introduction of covariates using the same variables as those previously described, along with the alternate introduction of neurocognitive covariates and clinical covariates (the various PANSS subscores). In these multiple analyses, every single social-cognitive tool was investigated separately, as we were interested in making recommendations about their single-use in a clinical context beyond usually available information such as clinical and neurocognitive profile. Each tool’s predictive power for the functioning (PSP) and QoL (S-QoL) at one year was explored similarly, except that the dependent outcome variables were taken at follow-up.

Standardized coefficients ß were determined for all regression analyses. For all results, positive ß and t-statistics indicated that better social cognitive performance was associated with a better outcome. More details on the statistics and criteria for defining satisfactory characteristics can be found in Supplementary Information 3.

### Reporting summary

Further information on research design is available in the Nature Research Reporting Summary linked to this article.

## Supplementary information

Supplementary Information

Reporting Summary

## Data Availability

The data presented here are not openly available to protect patient privacy. Requests for these data should be made to the corresponding author, E.B.G.
