# Supplementary material for: Outcome prediction with a social cognitive battery: a multicenter longitudinal study
Source: NPJ Schizophr. 2021 May 26;7:30. doi: 10.1038/s41537-021-00160-5 (PMC8155046; doi:10.1038/s41537-021-00160-5)
Supplement: Supplementary file 1 — Supplementary Information [file 41537_2021_160_MOESM1_ESM.docx]

**Supplementary Informations**

*Outcome prediction with a social cognitive battery: a multicenter longitudinal study*

Table of contents

[Supplementary Table 1. Baseline comparisons between completers and non-completers (t-test or chi² tests according to the nature of the variable investigated) 3](#_Toc67056090)

[Supplementary Table 2. Neurocognitive performance 4](#_Toc67056091)

[Supplementary Table 3. Change and Pearson correlations between inclusion and one-year assessment for clinical and functional variables in individuals who completed both visits. 5](#_Toc67056092)

[Supplementary Table 4. Standardized response means for neurocognition in individuals who completed both visits. 5](#_Toc67056093)

[Supplementary Table 5. Simple cross-sectional regressions of functioning (PSP) on social cognition at inclusion 6](#_Toc67056094)

[Supplementary Table 6. Simple cross-sectional regressions of functioning (PSP) on neurocognition at inclusion 6](#_Toc67056095)

[Supplementary Table 7. Simple cross-sectional regression of functioning (PSP) on clinical variables at inclusion 6](#_Toc67056096)

[Supplementary Table 8. Multiple cross-sectional regression of functioning (PSP) on social cognition beyond clinical symptoms at inclusion 7](#_Toc67056097)

[Supplementary Table 9. Multiple cross-sectional regression of functioning (PSP) on social cognition beyond neurocognition at inclusion 8](#_Toc67056098)

[Supplementary Table 10. Simple cross-sectional regression of quality of life (S-QoL) on social cognition at inclusion 8](#_Toc67056099)

[Supplementary Table 11. Simple cross-sectional regression of quality of life (S-QoL) on neurocognition 9](#_Toc67056100)

[Supplementary Table 12. Simple cross-sectional regression of quality of life (S-QoL) on clinical variables 9](#_Toc67056101)

[Supplementary Table 13. Multiple cross-sectional regression of quality of life (S-QoL) on social cognition beyond clinical variables 9](#_Toc67056102)

[Supplementary Table 14. Simple longitudinal regression of functioning (PSP) at follow-up on social cognition at inclusion 10](#_Toc67056103)

[Supplementary Table 15. Simple longitudinal regression of functioning (PSP) at follow-up on neurocognition at inclusion 10](#_Toc67056104)

[Supplementary Table 16. Simple longitudinal regression of functioning (PSP) at follow-up on clinical variables at inclusion 10](#_Toc67056105)

[Supplementary Table 17. Multiple regression of functioning (PSP) at follow-up on social cognition at inclusion beyond neurocognition at inclusion 11](#_Toc67056106)

[Supplementary Table 18. Multiple regression of functioning (PSP) at follow-up on social cognition at inclusion beyond clinical covariates at inclusion 11](#_Toc67056107)

[Supplementary Table 19. Simple longitudinal regression of quality of life (S-QoL) at follow-up on social cognition at inclusion 12](#_Toc67056108)

[Supplementary Table 20. Simple longitudinal regression of quality of life (S-QoL) at follow-up on neurocognition at inclusion 12](#_Toc67056109)

[Supplementary Information 1. Methods for the computation of the sensitivity of goal and belief attribution in SPEX-GBA (Social cognition, Perception, executive functions – Goal & Belief Attribution) 13](#_Toc67056110)

[Supplementary Information 2. Neuropsychological assessment 13](#_Toc67056111)

[Supplementary Information 3. Statistics 13](#_Toc67056112)

#

# Supplementary Table 1. Baseline comparisons between completers and non-completers (t-test or chi² tests according to the nature of the variable investigated)

| **Dimension** | **Variable** | **Version** | **Mean (sd) or % for completers** | **Mean (sd) or % for non-completers** | **Statistics** | **p** |
| --- | --- | --- | --- | --- | --- | --- |
| Clinical | Age at onset of illness (years) |  | 21.3(6.1) | 21.4(5) | t(130)=-0.1 | 0.886 |
|  | Duration of untreated psychosis (years) |  | 1.8(3.6) | 1.1(1.9) | t(124)=1.4 | 0.175 |
|  | Total duration of hospitalization (months) |  | 5.2(6.3) | 6.7(8.2) | t(111)=-1.1 | 0.28 |
|  | PANSS Positive |  | 9.8(4.2) | 8.8(4.4) | t(139)=1.4 | 0.17 |
|  | PANSS Negative |  | 16.8(6.7) | 16.2(7.5) | t(139)=0.5 | 0.631 |
|  | PANSS Disorganization |  | 8.3(3.6) | 7.5(3) | t(139)=1.5 | 0.135 |
|  | PANSS Excitation |  | 5.6(2.4) | 5.3(2.2) | t(139)=0.7 | 0.473 |
|  | PANSS Depression |  | 7.1(3.2) | 6.9(3.4) | t(139)=0.4 | 0.718 |
|  | Antipsychotics (chlorpromazine equivalents mg/24h) |  | 412.9(335.7) | 507.7(428.1) | t(117)=-1.3 | 0.182 |
|  | Males |  | 77.2 | 78.1 | χ²=0 | 1 |
|  | Age (years) |  | 31.7(8.8) | 31(7.4) | t(141)=0.4 | 0.655 |
|  | Educational level (years) |  | 12.8(2.2) | 12.3(2.7) | t(141)=1 | 0.297 |
|  | Premorbid IQ |  | 104.5(6.9) | 103.4(9.3) | t(137)=0.8 | 0.412 |
|  | Schizophrenia vs. Schizo-affective disorder |  | 74.7 | 70.3 | χ²=0.2 | 0.693 |
|  | CGI severity |  | 4.2(1.4) | 4(1.5) | t(137)=1 | 0.308 |
|  | PSP |  | 51.9(16.9) | 53.6(18.4) | t(132)=-0.6 | 0.57 |
|  | S-QoL |  | 48.3(19.4) | 52.5(20.1) | t(139)=-1.3 | 0.21 |
| Neurocognition | Speed of processing |  | -0.8(0.7) | -0.8(0.8) | t(138)=0.3 | 0.793 |
|  | Attention/vigilance |  | -0.3(0.5) | -0.4(0.5) | t(135)=0.2 | 0.867 |
|  | Working memory |  | -0.4(0.7) | -0.6(0.7) | t(136)=1.4 | 0.166 |
|  | Memory |  | -0.8(0.9) | -1(0.9) | t(137)=1 | 0.336 |
|  | Reasoning |  | -0.3(0.8) | -0.5(0.9) | t(135)=1.4 | 0.151 |
|  | Executive functions |  | -1(1.3) | -1.1(1.7) | t(136)=0.4 | 0.694 |
| Social cognition | TREF |  | 35.4(6.4) | 36.3(5.1) | t(112)=-0.8 | 0.437 |
|  | V-SIR |  | 17.4(6) | 17.5(5.9) | t(138)=-0.1 | 0.957 |
|  | QCAE affective |  | 2.3(0.4) | 2.2(0.4) | t(135)=1.4 | 0.179 |
|  | QCAE cognitive |  | 2.3(0.4) | 2.3(0.5) | t(135)=0.5 | 0.61 |
|  | SPEX- BA | 1 | 1.2(1.4) | 1.5(1.1) | t(49)=-0.9 | 0.366 |
|  | SPEX- BA | 2 | 1.2(1.1) | 1(1.3) | t(49)=0.5 | 0.618 |
|  | SPEX-GA | 1 | 1.3(1.4) | 1.5(1.3) | t(49)=-0.5 | 0.606 |
|  | SPEX-GA | 2 | 1.3(1.1) | 1.3(0.9) | t(49)=0 | 0.992 |
|  | V-Comics | 1 | 11.8(2.2) | 11.5(2.2) | t(70)=0.4 | 0.663 |
|  | V-Comics | 2 | 12.4(1.7) | 12.6(1.8) | t(69)=-0.4 | 0.656 |

*PANSS: Positive and Negative Syndrome Scale, CGI severity:* *Clinical Global Impression-Severity Scale, PSP: Personal and Social Performance Scale, S-QoL: Schizophrenia Quality of Life Questionnaire, TREF: Test de Reconnaissance des Émotions Faciales - Facial Emotions Recognition Task, V-SIR: Versailles-Situational Intention Reading, QCAE: Questionnaire of Cognitive and Affective Empathy, SPEX-BA & GA: Social cognition, Perception, eXecutive functions – Belief & Goal Attribution sensitivities, V-Comics: Versailles Intention Attribution Task*

# Supplementary Table 2. Neurocognitive performance

| Domain | Test | Variable | Mean | SD | Min | Max | N |
| --- | --- | --- | --- | --- | --- | --- | --- |
| Processing speed |  |  | -0.8 | 0.7 |  |  |  |
|  | Digit Symbol Coding / Coding | Number of correct symbols | -1.1 | 1 | -3 | 1.3 | 135 |
|  | TMT | Time to complete part A | -0.5 | 1.1 | -3.9 |  | 139 |
|  | Verbal fluency | Number of words beginning with P | -0.6 | 1 | -2.7 | 2.8 | 139 |
|  |  | Number of words in the animal category | -1 | 1 | -3.2 | 2.2 | 139 |
| Attention/vigilance |  |  | -0.4 | 0.5 |  |  |  |
|  | CPT-IP | Sensitivity (d’) | -1.1 | 1 | -3.1 | 1.2 | 118 |
|  | TAP-alertness | SD of reaction times without alert | -0.2 | 0.3 | -0.9 | 0.4 | 108 |
|  |  | SD of reaction times with alert | -0.1 | 0.3 | -0.9 | 0.5 | 108 |
|  |  | Phasic alert index | 0 | 0.3 | -0.6 | 0.9 | 108 |
|  | TAP-flexibility | Number of errors | 0 | 0.3 | -0.7 | 0.3 | 107 |
|  |  | Global performance index | -0.2 | 0.2 | -0.9 | 0.2 | 107 |
|  | TAP-divided attention | Number of errors | -0.1 | 0.3 | -0.9 | 0.2 | 110 |
|  |  | Number of omissions | -0.2 | 0.3 | -0.9 | 0.2 | 110 |
|  | TAP - Go / No Go 1 | SD or reaction times | -0.2 | 0.3 | -0.8 | 0.5 | 98 |
|  | TAP - Go / No Go 2 | SD of reaction times | -0.2 | 0.2 | -0.8 | 0.3 | 108 |
| Working memory |  |  | -0.5 | 0.7 |  |  |  |
|  | Digit Span | Number of correctly remembered digits in the forward condition | -0.2 | 1 | -2.6 | 2.8 | 137 |
|  |  | Number of correctly remembered digits in the backward condition | -0.4 | 0.9 | -2.3 | 2.9 | 137 |
|  | Arithmetic | Total score | -0.7 | 1.1 | -2.7 | 2.3 | 136 |
|  | Letter number sequencing | Total score | -0.7 | 0.8 | -2.7 | 1.3 | 85 |
| Memory |  |  | -0.9 | 0.9 |  |  |  |
|  | CVLT | Immediate recall | -1.5 | 1.5 | -5.1 | 1.4 | 133 |
|  |  | Short-delay free recall | -1.1 | 1.1 | -4.8 | 1.6 | 133 |
|  |  | Long-delay free recall | -1.1 | 1.2 | -4.2 | 1.6 | 133 |
|  |  | Total recognition | -0.3 | 1.3 | -2.6 | 1.2 | 133 |
|  | Doors test | Number of correctly recognized doors in condition A & B | -1.2 | 1 | -2.3 | 2 | 137 |
|  | Information | Total score | -0.2 | 1.1 | -2.3 | 2 | 137 |
| Reasoning |  |  | -0.4 | 0.9 |  |  |  |
|  | Matrices | Total score | -0.3 | 1.1 | -3 | 2.3 | 137 |
|  | Picture completion | Total score | -0.7 | 1 | -3 | 2 | 135 |
|  | Similarities | Total score | -0.1 | 1.1 | -2.7 | 2 | 137 |
| Executive functions |  |  | -1 | 1.5 |  |  |  |
|  | TMT | Time to complete part B | -1.2 | 2.1 | -12.1 | 1.4 | 136 |
|  | Multiple Errands Test | Total Errors Score | -0.8 | 1.1 | -2.9 | 0.7 | 125 |

Acknowledgments: all performance measurements are expressed as standard deviations from the norm.

TMT: Trail Making Test, CPT-IP: Continuous Performance Test-identical pairs version, TAP: Tests of Attentional Performance, CVLT: California Verbal Learning test.

# Supplementary Table 3. Change and Pearson correlations between inclusion and one-year assessment for clinical and functional variables in individuals who completed both visits.

| **Variable** | **Inclusion** | | **12 months** | | **Change** | | **Correlation** | | |
| --- | --- | --- | --- | --- | --- | --- | --- | --- | --- |
|  |  | |  | | **Statistics**  **(paired-t)** | **p** | **r** | **Statistics** | **p** |
|  | **Mean** | **SD** | **Mean** | **SD** |  |  |  |  |  |
| PANSS Positive | 9.3 | 4.3 | 8.4 | 3.4 | t(76) = 4.1 | **< 0.001** | 0.67 | t(75)=7.9 | **< 0.001** |
| PANSS Negative | 16.5 | 7 | 14.6 | 6.7 | t(76) = 3.3 | **0.002** | 0.53 | t(75)=5.4 | **< 0.001** |
| PANSS Disorganization | 8 | 3.4 | 7.2 | 3.8 | t(76) = 2.6 | **0.011** | 0.46 | t(75)=4.5 | **< 0.001** |
| PANSS Excitation | 5.4 | 2.3 | 5.2 | 2 | t(76) = 1.4 | 0.173 | 0.53 | t(75)=5.5 | **< 0.001** |
| PANSS Depression | 7 | 3.3 | 6.4 | 2.9 | t(76) = 2.1 | **0.042** | 0.47 | t(75)=4.7 | **< 0.001** |
| CGI-S | 4.1 | 1.4 | 3.7 | 1.6 | t(75) = 4 | **< 0.001** | 0.66 | t(74)=7.6 | **< 0.001** |
| PSP | 52.6 | 17.5 | 58 | 16.6 | t(74) = -4.4 | **< 0.001** | 0.74 | t(73)=9.4 | **< 0.001** |
| SQOL | 50.1 | 19.8 | 54 | 17 | t(73) = -3.5 | **0.001** | 0.64 | t(72)=7.1 | **< 0.001** |

PANSS: Positive and Negative Syndrome Scale, CGI: Clinical Global Impression-Severity Scale, PSP: Personal and Social Performance Scale, SQOL: Schizophrenia Quality of Life Questionnaire.

# Supplementary Table 4. Standardized response means for neurocognition in individuals who completed both visits.

| **Variable** | **Inclusion** | | **Follow-up** | | **SRM** |
| --- | --- | --- | --- | --- | --- |
|  | **Mean** | **SD** | **Mean** | **SD** |  |
| Digit Span - Forward | -0.1 | 1 | -0.03 | 0.8 | 0.07 |
| Digit Span - Backward | -0.19 | 0.89 | -0.09 | 0.92 | 0.12 |
| TMT A | -0.44 | 0.97 | -0.16 | 0.88 | 0.39 |
| TMT B | -1.11 | 2.1 | -0.74 | 1.38 | 0.22 |
| CPT-IP | -0.92 | 0.97 | -0.74 | 0.95 | 0.28 |
| Phonemic fluency | -0.7 | 1.01 | -0.63 | 1.04 | 0.08 |
| Semantic fluency | -1 | 0.93 | -0.99 | 0.99 | 0.02 |
| TAP-Alertness - SD of reaction times without alert | -0.13 | 0.31 | -0.16 | 0.29 | -0.15 |
| TAP-Alertness - SD of reaction times with alert | -0.06 | 0.26 | -0.12 | 0.24 | -0.25 |
| TAP-Alertness - Phasic alert index | 0.02 | 0.23 | -0.02 | 0.19 | -0.14 |
| TAP-flexibility- Number of errors | -0.02 | 0.27 | 0.06 | 0.21 | 0.29 |
| TAP-flexibility- global performance index | -0.16 | 0.26 | -0.05 | 0.22 | 0.59 |
| TAP-divided attention-number of errors | -0.09 | 0.24 | -0.16 | 0.28 | -0.32 |
| TAP-divided attention- Number of omissions | -0.22 | 0.29 | -0.17 | 0.27 | 0.19 |
| TAP - Go / No Go 1 | -0.14 | 0.25 | -0.13 | 0.26 | 0.04 |
| TAP - Go / No Go 2 | -0.14 | 0.2 | -0.13 | 0.28 | 0.04 |

SRM: Standardized response mean, TMT: Trail Making Test, CPT-IP: Continuous Performance Test-identical pairs version, TAP: Tests of Attentional Performance.

# Supplementary Table 5. Simple cross-sectional regressions of functioning (PSP) on social cognition at inclusion

| **Dimension** | **Variable** | **Version** | **Standardized coefficient ß** | **Standard Error** | **Statistics** | **p** | **fmi** | **λ** |
| --- | --- | --- | --- | --- | --- | --- | --- | --- |
| Facial emotion recognition | TREF |  | 0.32 | 0.08 | t(119.8) = 3.7 | **< 0.001** | 0.13 | 0.11 |
| Theory of mind | V-SIR |  | 0.29 | 0.08 | t(133.2) = 3.5 | **0.001** | 0.05 | 0.04 |
|  | SPEX-BA | 1 | 0.15 | 0.13 | t(51.9) = 1.2 | 0.252 | 0.23 | 0.2 |
|  | SPEX-BA | 2 | 0.08 | 0.14 | t(46.3) = 0.5 | 0.595 | 0.29 | 0.26 |
|  | SPEX-GA | 1 | 0.19 | 0.12 | t(54.5) = 1.6 | 0.119 | 0.2 | 0.17 |
|  | SPEX-GA | 2 | 0.03 | 0.16 | t(43) = 0.2 | 0.868 | 0.33 | 0.3 |
|  | V-Comics | 1 | 0.38 | 0.1 | t(61.7) = 3.6 | **0.001** | 0.11 | 0.09 |
|  | V-Comics | 2 | 0.25 | 0.14 | t(67) = 1.8 | 0.076 | 0.03 | 0 |
| Self-reported empathy | QCAE affective |  | 0.13 | 0.09 | t(132.9) = 1.5 | 0.138 | 0.05 | 0.04 |
|  | QCAE cognitive |  | -0.05 | 0.09 | t(132.6) = -0.6 | 0.529 | 0.06 | 0.04 |

Fmi: fraction of missing information, λ: fraction of total variance due to missing data, TREF: *Test de Reconnaissance des Émotions Faciales* - Facial Emotion Recognition Test, V-SIR: Versailles Situational Intention Reading, SPEX-BA & GA: sensitivity of Social cognition, Perception, eXecutive functions – Belief & Goal Attribution, V-Comics: Versailles Intention Attribution Task, QCAE: Questionnaire of Cognitive and Affective Empathy. The signs of the ß and t statistics were reversed for V-SIR.

# Supplementary Table 6. Simple cross-sectional regressions of functioning (PSP) on neurocognition at inclusion

| **Neurocognitive measure** | **Standardized coefficient ß** | **Standard Error** | **Statistics** | **p** | **fmi** | **λ** |
| --- | --- | --- | --- | --- | --- | --- |
| Speed of processing | 0.27 | 0.08 | t(135.5) = 3.3 | **0.001** | 0.04 | 0.02 |
| Attention | 0.2 | 0.09 | t(124.8) = 2.3 | **0.023** | 0.1 | 0.09 |
| Working memory | 0.21 | 0.08 | t(129.8) = 2.5 | **0.013** | 0.07 | 0.06 |
| Memory | 0.23 | 0.08 | t(132.4) = 2.8 | **0.007** | 0.06 | 0.04 |
| Reasoning | 0.32 | 0.08 | t(133.5) = 3.9 | **< 0.001** | 0.05 | 0.04 |
| Executive functions | 0.34 | 0.08 | t(132.3) = 4.2 | **< 0.001** | 0.06 | 0.04 |

Fmi: fraction of missing information, λ: fraction of total variance due to missing data.

# Supplementary Table 7. Simple cross-sectional regression of functioning (PSP) on clinical variables at inclusion

| **Clinical variable** | **Standardized coefficient ß** | **Standard Error** | **Statistics** | **p** | **fmi** | **λ** |
| --- | --- | --- | --- | --- | --- | --- |
| Positive | -0.38 | 0.08 | t(136.5) = -4.8 | **< 0.001** | 0.03 | 0.02 |
| Negative | -0.43 | 0.08 | t(137.9) = -5.7 | **< 0.001** | 0.02 | 0.01 |
| Disorganization | -0.41 | 0.08 | t(137.2) = -5.3 | **< 0.001** | 0.03 | 0.01 |
| Excitation | -0.38 | 0.08 | t(136.1) = -4.9 | **< 0.001** | 0.03 | 0.02 |
| Depression | -0.25 | 0.08 | t(128.9) = -3 | **0.003** | 0.08 | 0.06 |

Fmi: fraction of missing information, λ: fraction of total variance due to missing data.

#

# Supplementary Table 8. Multiple cross-sectional regression of functioning (PSP) on social cognition beyond clinical symptoms at inclusion

| **Social cognition**  **Dimension Variable** | | **Clinical covariate**  **(PANSS)** | **Standardized coefficient ß** | **Standard Error** | **Statistics** | **p** | **fmi** | **λ** |
| --- | --- | --- | --- | --- | --- | --- | --- | --- |
| Facial emotion recognition | TREF | Positive | 0.28 | 0.08 | t(114.3) = 3.4 | **0.001** | 0.15 | 0.14 |
|  |  | Negative | 0.22 | 0.08 | t(110.8) = 2.6 | **0.01** | 0.17 | 0.15 |
|  |  | Disorganization | 0.21 | 0.09 | t(114.9) = 2.4 | **0.018** | 0.15 | 0.13 |
|  |  | Excitation | 0.26 | 0.08 | t(118.4) = 3.3 | **0.001** | 0.13 | 0.11 |
|  |  | Depression | 0.34 | 0.08 | t(120.7) = 4.2 | **< 0.001** | 0.12 | 0.1 |
| Theory of mind | V-SIR | Positive | 0.26 | 0.08 | t(131.1) = 3.4 | **0.001** | 0.06 | 0.05 |
|  |  | Negative | 0.19 | 0.08 | t(129.4) = 2.4 | **0.016** | 0.07 | 0.06 |
|  |  | Disorganization | 0.18 | 0.08 | t(131.9) = 2.1 | **0.034** | 0.05 | 0.04 |
|  |  | Excitation | 0.29 | 0.08 | t(131.4) = 3.8 | **< 0.001** | 0.06 | 0.04 |
|  |  | Depression | 0.31 | 0.08 | t(130.8) = 4 | **< 0.001** | 0.06 | 0.05 |
|  | V-Comics version 1 | Positive | 0.3 | 0.1 | t(58.7) = 3 | **0.004** | 0.14 | 0.11 |
|  |  | Negative | 0.31 | 0.1 | t(59.6) = 3.2 | **0.002** | 0.13 | 0.1 |
|  |  | Disorganization | 0.29 | 0.11 | t(59.7) = 2.8 | **0.008** | 0.13 | 0.1 |
|  |  | Excitation | 0.3 | 0.1 | t(59) = 2.9 | **0.005** | 0.14 | 0.11 |
|  |  | Depression | 0.39 | 0.1 | t(60.3) = 4 | **< 0.001** | 0.12 | 0.09 |

PANSS: Positive and Negative Syndrome Scale, fmi: fraction of missing information, λ: fraction of total variance due to missing data, TREF: *Test de Reconnaissance des Émotions Faciales* - Facial Emotion Recognition Test, V-SIR: Versailles Situational Intention Reading, V-Comics: Versailles Intention Attribution Task. The signs of the ß and t statistics were reversed for V-SIR.

# Supplementary Table 9. Multiple cross-sectional regression of functioning (PSP) on social cognition beyond neurocognition at inclusion

| **Social Cognition**  **Dimension Variable** | | **Neurocognitive covariate** | **Standardized coefficient ß** | | **Standard error** | **Statistics** | **p** | | **fmi** | **λ** | |
| --- | --- | --- | --- | --- | --- | --- | --- | --- | --- | --- | --- |
| Facial emotion recognition | TREF | Processing speed | 0.27 | 0.09 | | t(116.9) = 3.1 | | **0.003** | 0.14 | | 0.12 |
|  |  | Attention | 0.29 | 0.09 | | t(118.4) = 3.4 | | **0.001** | 0.13 | | 0.11 |
|  |  | Working memory | 0.28 | 0.09 | | t(120.9) = 3.1 | | **0.003** | 0.12 | | 0.1 |
|  |  | Memory | 0.27 | 0.09 | | t(114.3) = 2.9 | | **0.005** | 0.15 | | 0.14 |
|  |  | Reasoning | 0.21 | 0.1 | | t(112.2) = 2.2 | | **0.03** | 0.16 | | 0.15 |
|  |  | Executive functions | 0.23 | 0.09 | | t(114.7) = 2.6 | | **0.011** | 0.15 | | 0.13 |
| Theory of mind | V-SIR | Processing speed | 0.21 | 0.09 | | t(132.6) = 2.3 | | **0.022** | 0.05 | | 0.04 |
|  |  | Attention | 0.27 | 0.08 | | t(132.3) = 3.3 | | **0.001** | 0.05 | | 0.04 |
|  |  | Working memory | 0.24 | 0.09 | | t(127.1) = 2.6 | | **0.011** | 0.08 | | 0.07 |
|  |  | Memory | 0.23 | 0.09 | | t(130.9) = 2.5 | | **0.015** | 0.06 | | 0.05 |
|  |  | Reasoning | 0.16 | 0.1 | | t(131.6) = 1.5 | | 0.125 | 0.06 | | 0.04 |
|  |  | Executive functions | 0.17 | 0.09 | | t(131.2) = 1.9 | | 0.065 | 0.06 | | 0.04 |
|  | V-Comics | Processing speed | 0.31 | 0.11 | | t(60.7) = 2.8 | | **0.008** | 0.12 | | 0.09 |
|  | Version 1 | Attention | 0.36 | 0.11 | | t(61.5) = 3.4 | | **0.001** | 0.1 | | 0.08 |
|  |  | Working memory | 0.3 | 0.12 | | t(62.5) = 2.6 | | **0.013** | 0.09 | | 0.06 |
|  |  | Memory | 0.28 | 0.12 | | t(59.3) = 2.3 | | **0.027** | 0.13 | | 0.1 |
|  |  | Reasoning | 0.24 | 0.14 | | t(58.6) = 1.8 | | 0.082 | 0.14 | | 0.11 |
|  |  | Executive functions | 0.26 | 0.11 | | t(59.5) = 2.3 | | **0.026** | 0.13 | | 0.1 |

Fmi: fraction of missing information, λ: fraction of total variance due to missing data TREF: Test de Reconnaissance des Émotions Faciales - Facial Emotion Recognition Test, V-SIR: Versailles Situational Intention Reading, V-Comics: Versailles Intention Attribution Task. The signs of the ß and t statistics were reversed for V-SIR.

# Supplementary Table 10. Simple cross-sectional regression of quality of life (S-QoL) on social cognition at inclusion

| **Dimension** | **Variable** | **Version** | **Standardized coefficient ß** | **Standard Error** | **Statistics** | **p** | **fmi** | **λ** |
| --- | --- | --- | --- | --- | --- | --- | --- | --- |
| Facial emotion recognition | TREF |  | -0.01 | 0.09 | t(125.6) = -0.1 | 0.944 | 0.1 | 0.08 |
| Theory of mind | V-SIR |  | -0.1 | 0.08 | t(137.1) = -1.1 | 0.262 | 0.03 | 0.01 |
|  | SPEX- BA | 1 | -0.06 | 0.12 | t(54.7) = -0.5 | 0.601 | 0.2 | 0.17 |
|  | SPEX- BA | 2 | 0.09 | 0.14 | t(52.2) = 0.6 | 0.523 | 0.22 | 0.19 |
|  | SPEX-GA | 1 | -0.16 | 0.11 | t(55.3) = -1.4 | 0.168 | 0.19 | 0.16 |
|  | SPEX-GA | 2 | 0.09 | 0.16 | t(48.1) = 0.6 | 0.581 | 0.27 | 0.24 |
|  | V-Comics | 1 | -0.06 | 0.11 | t(67.5) = -0.6 | 0.553 | 0.04 | 0.01 |
|  | V-Comics | 2 | 0.13 | 0.15 | t(66.6) = 0.9 | 0.392 | 0.04 | 0.01 |
| Self-reported empathy | QCAE affective |  | 0.17 | 0.08 | t(134.3) = 2 | **0.044** | 0.05 | 0.03 |
|  | QCAE cognitive |  | -0.16 | 0.09 | t(131.7) = -1.8 | 0.07 | 0.06 | 0.05 |

Fmi: fraction of missing information, λ: fraction of total variance due to missing data, TREF: *Test de Reconnaissance des Émotions Faciales* - Facial Emotion Recognition Test, V-SIR: Versailles Situational Intention Reading, SPEX-BA & GA: sensitivity of Social cognition, Perception, eXecutive functions – Belief & Goal Attribution, V-Comics: Versailles Intention Attribution Task, QCAE: Questionnaire of Cognitive and Affective Empathy. The signs of the ß and t statistics were reversed for V-SIR.

# Supplementary Table 11. Simple cross-sectional regression of quality of life (S-QoL) on neurocognition

| **Neurocognitive measure** | | **Standardized coefficient ß** | **Standard Error** | **Statistics** | **p** | **fmi** | **λ** |
| --- | --- | --- | --- | --- | --- | --- | --- |
| Speed of processing | 0.09 | | 0.09 | t(133.6) = 1 | 0.311 | 0.05 | 0.04 |
| Attention | -0.04 | | 0.09 | t(134) = -0.5 | 0.629 | 0.05 | 0.03 |
| Working memory | -0.06 | | 0.09 | t(131.1) = -0.7 | 0.496 | 0.06 | 0.05 |
| Memory | -0.09 | | 0.09 | t(130.5) = -1 | 0.31 | 0.07 | 0.05 |
| Reasoning | -0.05 | | 0.09 | t(135) = -0.6 | 0.581 | 0.04 | 0.03 |
| Executive functions | 0 | | 0.09 | t(133.1) = 0 | 0.99 | 0.05 | 0.04 |

Fmi: fraction of missing information, λ: fraction of total variance due to missing data.

# Supplementary Table 12. Simple cross-sectional regression of quality of life (S-QoL) on clinical variables

| **Clinical variable (PANSS)** | **Standardized coefficient ß** | **Standard Error** | **Statistics** | **p** | **fmi** | **λ** |
| --- | --- | --- | --- | --- | --- | --- |
| Positive | -0.17 | 0.08 | t(137.6) = -2 | **0.048** | 0.02 | 0.01 |
| Negative | -0.26 | 0.08 | t(137.4) = -3.1 | **0.002** | 0.03 | 0.01 |
| Disorganization | -0.03 | 0.09 | t(136.1) = -0.4 | 0.704 | 0.03 | 0.02 |
| Excitation | -0.09 | 0.08 | t(137) = -1 | 0.3 | 0.03 | 0.01 |
| Depression | -0.53 | 0.07 | t(135.7) = -7.3 | **< 0.001** | 0.04 | 0.02 |

PANSS: Positive and Negative Syndrome Scale, fmi: fraction of missing information, λ: fraction of total variance due to missing data.

# Supplementary Table 13. Multiple cross-sectional regression of quality of life (S-QoL) on social cognition beyond clinical variables

| **Social Cognition**  **Dimension Variable** | | **Clinical covariate** | **Standardized coefficient ß** | | **Standard Error** | **Statistics** | **p** | | **fmi** | **λ** | |
| --- | --- | --- | --- | --- | --- | --- | --- | --- | --- | --- | --- |
| Self-reported empathy | QCAE affective | Positive | 0.16 | 0.08 | | t(133.8) = 1.9 | | 0.055 | 0.04 | | 0.03 |
|  |  | Negative | 0.15 | 0.08 | | t(132) = 1.8 | | 0.07 | 0.05 | | 0.04 |
|  |  | Disorganization | 0.17 | 0.09 | | t(133.1) = 2 | | **0.049** | 0.05 | | 0.03 |
|  |  | Excitation | 0.17 | 0.08 | | t(133.4) = 2 | | **0.048** | 0.05 | | 0.03 |
|  |  | Depression | 0.08 | 0.07 | | t(133.5) = 1.1 | | 0.258 | 0.04 | | 0.03 |

Fmi: fraction of missing information, λ: fraction of total variance due to missing data, QCAE: Questionnaire of Cognitive and Affective Empathy.

# Supplementary Table 14. Simple longitudinal regression of functioning (PSP) at follow-up on social cognition at inclusion

| **Dimension** | **Variable** | **Version** | **Standardized coefficient ß** | **Standard Error** | **Statistic** | **p** | **fmi** | **λ** |
| --- | --- | --- | --- | --- | --- | --- | --- | --- |
| Facial emotion recognition | TREF |  | 0.2 | 0.1 | t(69.8) = 1.9 | 0.058 | 0.39 | 0.37 |
| Theory of mind | V-SIR |  | 0.17 | 0.1 | t(84.6) = 1.8 | 0.079 | 0.31 | 0.29 |
|  | SPEX-BA | 1 | 0.22 | 0.14 | t(44.9) = 1.6 | 0.112 | 0.32 | 0.29 |
|  | SPEX-BA | 2 | 0.13 | 0.16 | t(32) = 0.8 | 0.424 | 0.48 | 0.45 |
|  | SPEX-GA | 1 | 0.25 | 0.13 | t(41.9) = 2 | 0.057 | 0.35 | 0.32 |
|  | SPEX-GA | 2 | 0.15 | 0.16 | t(40.5) = 1 | 0.335 | 0.37 | 0.33 |
|  | V-Comics | 1 | 0.33 | 0.13 | t(40.3) = 2.6 | **0.013** | 0.37 | 0.34 |
|  | V-Comics | 2 | 0.09 | 0.16 | t(47.2) = 0.6 | 0.557 | 0.28 | 0.25 |
| Self-reported empathy | QCAE affective |  | -0.11 | 0.1 | t(92.6) = -1.2 | 0.243 | 0.26 | 0.25 |
|  | QCAE cognitive |  | -0.12 | 0.11 | t(65.4) = -1.1 | 0.267 | 0.42 | 0.4 |

Fmi: fraction of missing information, λ: fraction of total variance due to missing data, TREF: *Test de Reconnaissance des Émotions Faciales* - Facial Emotion Recognition Test, V-SIR: Versailles Situational Intention Reading, SPEX-BA & GA: sensitivity of Social cognition, Perception, eXecutive functions – Belief & Goal Attribution, V-Comics: Versailles Intention Attribution Task, QCAE: Questionnaire of Cognitive and Affective Empathy. The signs of the ß and t statistics were reversed for V-SIR.

# Supplementary Table 15. Simple longitudinal regression of functioning (PSP) at follow-up on neurocognition at inclusion

| **Neurocognitive measure** | **Standardized coefficient ß** | **Standard Error** | **Statistics** | **p** | **fmi** | **λ** |
| --- | --- | --- | --- | --- | --- | --- |
| Speed of processing | 0.26 | 0.11 | t(65.2) = 2.4 | **0.018** | 0.42 | 0.4 |
| Attention | 0.23 | 0.1 | t(79.4) = 2.3 | **0.022** | 0.33 | 0.32 |
| Working memory | 0.19 | 0.1 | t(89.8) = 1.9 | 0.055 | 0.28 | 0.26 |
| Memory | 0.14 | 0.11 | t(63.5) = 1.3 | 0.187 | 0.43 | 0.41 |
| Reasoning | 0.28 | 0.1 | t(65.8) = 2.7 | **0.008** | 0.42 | 0.4 |
| Executive functions | 0.38 | 0.09 | t(81.3) = 4.1 | **< 0.001** | 0.32 | 0.31 |

Fmi: fraction of missing information, λ: fraction of total variance due to missing data.

# Supplementary Table 16. Simple longitudinal regression of functioning (PSP) at follow-up on clinical variables at inclusion

| **Clinical variable at inclusion** | **Standardized coefficient ß** | **Standard Error** | **Statistics** | **p** | **fmi** | **λ** |
| --- | --- | --- | --- | --- | --- | --- |
| Positive | -0.46 | 0.09 | t(72.9) = -4.9 | **< 0.001** | 0.37 | 0.35 |
| Negative | -0.18 | 0.1 | t(79.4) = -1.8 | 0.082 | 0.33 | 0.32 |
| Disorganization | -0.38 | 0.09 | t(91.5) = -4.2 | **< 0.001** | 0.27 | 0.25 |
| Excitation | -0.32 | 0.09 | t(101.9) = -3.6 | **< 0.001** | 0.22 | 0.2 |
| Depression | -0.2 | 0.1 | t(78.6) = -2 | **0.047** | 0.34 | 0.32 |
| Functioning | 0.62 | 0.09 | t(58.7) = 7 | **< 0.001** | 0.46 | 0.45 |
| Quality of life | 0.21 | 0.1 | t(85.9) = 2.2 | **0.03** | 0.3 | 0.28 |

Fmi: fraction of missing information, λ: fraction of total variance due to missing data.

# Supplementary Table 17. Multiple regression of functioning (PSP) at follow-up on social cognition at inclusion beyond neurocognition at inclusion

| **Dimension** | **Variable** | **Neurocognitive covariate** | **Standardized coefficient ß** | **Standard Error** | | **Statistics** | **p** | | **fmi** | **λ** |
| --- | --- | --- | --- | --- | --- | --- | --- | --- | --- | --- |
| Theory of mind | V-Comics version 1 | Speed of processing | 0.23 | 0.14 | t(34.3) = 1.7 | | 0.108 | 0.45 | | 0.42 |
|  |  | Attention | 0.3 | 0.13 | t(38.9) = 2.3 | | **0.026** | 0.39 | | 0.36 |
|  |  | Working memory | 0.23 | 0.14 | t(39.7) = 1.6 | | 0.115 | 0.38 | | 0.34 |
|  |  | Memory | 0.25 | 0.14 | t(45.6) = 1.8 | | 0.077 | 0.3 | | 0.27 |
|  |  | Reasoning | 0.1 | 0.15 | t(42.3) = 0.7 | | 0.514 | 0.34 | | 0.31 |
|  |  | Executive functions | 0.15 | 0.14 | t(35.1) = 1.1 | | 0.287 | 0.44 | | 0.41 |

Fmi: fraction of missing information, λ: fraction of total variance due to missing data, TREF: *Test de Reconnaissance des Émotions Faciales* - Facial Emotion Recognition Test, V-Comics: Versailles Intention Attribution Task.

# Supplementary Table 18. Multiple regression of functioning (PSP) at follow-up on social cognition at inclusion beyond clinical covariates at inclusion

| **Dimension** | **Variable** | **Clinical covariate** | **Standardized coefficient ß** | **Standard Error** | **Statistics** | **p** | **fmi** | **λ** |
| --- | --- | --- | --- | --- | --- | --- | --- | --- |
| Theory of mind | V-Comics version 1 | Positive | 0.23 | 0.12 | t(34.2) = 1.9 | 0.065 | 0.45 | 0.42 |
|  |  | Negative | 0.3 | 0.13 | t(37.8) = 2.3 | **0.026** | 0.4 | 0.37 |
|  |  | Disorganization | 0.23 | 0.13 | t(37) = 1.8 | 0.083 | 0.41 | 0.38 |
|  |  | Excitation | 0.25 | 0.13 | t(36.3) = 2 | 0.055 | 0.42 | 0.39 |
|  |  | Depression | 0.34 | 0.13 | t(39.6) = 2.7 | **0.01** | 0.38 | 0.35 |
|  |  | Functioning | 0.12 | 0.13 | t(28.6) = 0.9 | 0.391 | 0.53 | 0.5 |
|  |  | Quality of life | 0.34 | 0.13 | t(39.6) = 2.7 | **0.011** | 0.38 | 0.35 |

V-Comics: Versailles Intention Attribution Task, Fmi: fraction of missing information, λ: fraction of total variance due to missing data.

# Supplementary Table 19. Simple longitudinal regression of quality of life (S-QoL) at follow-up on social cognition at inclusion

| **Dimension** | **Variable** | **Version** | **Standardized coefficient ß** | **Standard Error** | **Statistics** | **p** | **fmi** | **λ** |
| --- | --- | --- | --- | --- | --- | --- | --- | --- |
| Facial emotion recognition | TREF |  | -0.1 | 0.12 | t(53.3) = -0.8 | 0.416 | 0.5 | 0.48 |
| Theory of mind | V-SIR |  | -0.13 | 0.12 | t(50.7) = -1.1 | 0.269 | 0.52 | 0.5 |
|  | SPEX-BA | 1 | 0.03 | 0.14 | t(46.8) = 0.3 | 0.8 | 0.29 | 0.26 |
|  | SPEX-BA | 2 | 0.1 | 0.16 | t(35.4) = 0.7 | 0.518 | 0.43 | 0.4 |
|  | SPEX-GA | 1 | 0.08 | 0.14 | t(36) = 0.5 | 0.593 | 0.43 | 0.4 |
|  | SPEX-GA | 2 | 0.2 | 0.17 | t(37.1) = 1.2 | 0.231 | 0.41 | 0.38 |
|  | V-Comics | 1 | -0.11 | 0.15 | t(30.2) = -0.7 | 0.468 | 0.51 | 0.48 |
|  | V-Comics | 2 | 0 | 0.18 | t(33.2) = 0 | 0.989 | 0.46 | 0.43 |
| Self-reported empathy | QCAE affective |  | 0.02 | 0.11 | t(65.9) = 0.2 | 0.841 | 0.42 | 0.4 |
|  | QCAE cognitive |  | -0.13 | 0.13 | t(39.3) = -1 | 0.345 | 0.62 | 0.6 |

Fmi: fraction of missing information, λ: fraction of total variance due to missing data, TREF: *Test de Reconnaissance des Émotions Faciales* - Facial Emotion Recognition Test, V-SIR: Versailles Situational Intention Reading, SPEX-BA & GA: sensitivity of Social cognition, Perception, eXecutive functions – Belief & Goal Attribution, V-Comics: Versailles Intention Attribution Task, QCAE: Questionnaire of Cognitive and Affective Empathy. The signs of the ß and t statistics were reversed for V-SIR.

# Supplementary Table 20. Simple longitudinal regression of quality of life (S-QoL) at follow-up on neurocognition at inclusion

| **Neurocognitive measure** | **Standardized coefficient ß** | **Standard Error** | **Statistics** | **p** | **fmi** | **λ** |
| --- | --- | --- | --- | --- | --- | --- |
| Speed of processing | 0.03 | 0.13 | t(43.4) = 0.3 | 0.793 | 0.58 | 0.57 |
| Attention | 0.01 | 0.12 | t(54.3) = 0.1 | 0.924 | 0.5 | 0.48 |
| Working memory | -0.08 | 0.1 | t(72.2) = -0.8 | 0.419 | 0.38 | 0.36 |
| Memory | -0.14 | 0.12 | t(46.5) = -1.1 | 0.273 | 0.56 | 0.54 |
| Reasoning | -0.06 | 0.12 | t(51) = -0.5 | 0.601 | 0.52 | 0.5 |
| Executive functions | 0.05 | 0.12 | t(54.4) = 0.4 | 0.661 | 0.5 | 0.48 |

Fmi: fraction of missing information, λ: fraction of total variance due to missing data.

# Supplementary Information 1. Methods for the computation of the sensitivity of goal and belief attribution in SPEX-GBA (Social cognition, Perception, executive functions – Goal & Belief Attribution)

The sensitivity of goal and belief attribution was calculated from 12 items that assess the attribution of goals and beliefs with the following formula: d’=z(H)-z(F). For sensitivity of goal attribution, z(H) and z(F) represent the z-scores of the rate of correct responses under the "change seen" condition (4 items) and the rate of choosing the same response (incorrect) under the "no change" condition (4 items), respectively. For the sensitivity of belief attribution, z(H) and z(F) represent the z-scores of the rate of good responses in the condition of "change not seen" (4 items) and the rate of choice of the same response (incorrect) in the condition of "change not seen" (4 items), respectively.

# Supplementary Information 2. Neuropsychological assessment

The following tests were used, grouped into six domains:

1. **Speed of processing**, assessed using the Digit Symbol Coding subtest from the Wechsler Adult Intelligence Scale (WAIS) version III ^1^ or the Coding subtest from the WAIS-IV ^2^, TMT A (Trail Making Test, Part A) ^3^, and Verbal Fluency (semantic and phonemic) ^4^.
2. **Attention/vigilance**, assessed using the Continuous Performance Test-identical pairs version ^5^, alertness, flexibility, and divided attention, go-no go 1 & 2 subtests from the Tests of Attentional Performance ^6^.
3. **Working memory**, assessed using the digit span, arithmetic, and letter-number sequencing subtests from the WAIS.
4. **Memory**, assessed using the California Verbal Learning Test ^7^, the Door Test ^8^, and the information subtest from the WAIS-III or IV.
5. **Reasoning**, assessed using the matrices, picture completion, and similarities subtests from the WAIS-III or IV.
6. **Executive functions**, assessed using the TMT B ^3^, Multiple Errands Test ^9^.

Normalized z-scores were obtained with normative data from ^10–12^.

# Supplementary Information 3. Statistics

Each measure of social cognition was tested for:

- **Normality**: Measures with a coefficient of symmetry |skewness| < 1 were considered normal ^13^.
- **Outliers**, defined as values > 1.5 times the interquartile range above the upper quartile and below the lower quartile. In subsequent analyses, these values were replaced by the nearest non-suspect neighbors ^14^.
- **Ceiling or floor effects**, as the number of participants with the minimum or maximum score. If 15% of the evaluated sample obtains the highest/lowest possible score for a measure, the instrument suffers from a ceiling/floor effect ^15^.
- **Internal consistency**, assessed by Cronbach's alpha coefficient (α). The threshold at which this internal consistency was considered acceptable was set to 0.7 ^16^.
- **Sensitivity to change:** There is no standard procedure to calculate sensitivity to change, as current approaches vary ^17,18^. To estimate the sensitivity to change of the various social cognitive measures, we calculated the standardized response mean (SRM) as the mean of the change scores divided by the standard deviation of the change scores ^19^. This coefficient has been considered to be the most adequate index of sensitivity to change, as it incorporates the response variance in the denominator ^20^. It can be assessed without intervention between the different times of assessment ^21^, as in an observational design. The SRM is appropriate when the sample is expected to have similar magnitudes of change ^22^. Prior knowledge of the FACE-SCZ cohort allowed the hypothesis that the participants included in this study would show a global improvement in clinical and functional outcomes. These improvements were tested using paired-sample t-tests. The coefficients were interpreted as follows: SRM ≤ 0.1 = small sensitivity to change, 0.1 < SRM ≤ 0.5 = medium sensitivity to change, SRM > 0.5 = large sensitivity to change ^23,24^. A medium or large sensitivity to change was considered to be acceptable.

Simple cross-sectional relationships between social cognition and outcome measures (i.e., functioning and QoL) at inclusion were explored using successive univariate linear regressions. We tested whether the previously identified associations resisted the introduction of covariates by conducting multiple variable linear regression analyses using the same variables as those previously described, but alternately introducing the neurocognitive covariates (mean scores on the different neurocognitive dimensions) and clinical covariates (the different PANSS subscores). The incremental prediction was considered to be valid only if none of the introduced variables cancelled the relationship between social cognition and outcome. The predictive power of social cognition to functioning and QoL at one year was explored in a similar manner, except that dependent outcome variables were taken at one year.

**References**

1. Wechsler, D. *WAIS-III, Wechsler adult intelligence scale: Administration and scoring manual*. (Psychological Corporation, 1997).

2. Wechsler, D., Coalson, D. L. & Raiford, S. E. *WAIS-IV: Wechsler adult intelligence scale*. (Pearson San Antonio, TX, 2008).

3. Reitan, R. M. Validity of the Trail Making Test as an indicator of organic brain damage. *Percept Mot Ski.* 8, 271–276 (1958).

4. Lezak, M. D. *Neuropsychological assessment*. (Oxford University Press, USA, 2004).

5. Cornblatt, B. A., Risch, N. J., Faris, G., Friedman, D. & Erlenmeyer-Kimling, L. The Continuous Performance Test, identical pairs version (CPT-IP): I. New findings about sustained attention in normal families. *Psychiatry Res* 26, 223–238 (1988).

6. Zimmermann, P. & Fimm, B. A test battery for attentional performance. in *Applied neuropsychology of attention.* 110–151 (Psychology Press, 2002).

7. Delis, D. C. *CVLT-II: California verbal learning test: adult version*. (Psychological Corporation, 2000).

8. Baddeley, A. D., Emslie, H. & Nimmo-Smith, I. *Doors and people: a test of visual and verbal recall and recognition*. (Harcourt Assessment, 2006).

9. Shallice, T. & Burgess, P. W. Deficits in Strategy Application Following Frontal Lobe Damage in Man. *Brain* 114, 727–741 (1991).

10. Wechsler, D. *WAIS III: Echelle d’intelligence pour adultes*. (Paris, France: Les éditions du Centre de Psychologie appliquée (ECPA), 1997).

11. Wechsler, D. *WAIS-IV: échelle d’intelligence de Wechsler pour adultes*. (Pearson, 2010).

12. Poitrenaud, J., Deweer, B., Kalafat, M. & Van der Linden, M. *Adaptation en langue française du California Verbal Learning Test*. (Les Editions du Centre de Psychologie Appliquée, 2007).

13. Kerman, S. C. & McDonald, J. B. Skewness–kurtosis bounds for the skewed generalized T and related distributions. *Stat. Probab. Lett.* 83, 2129–2134 (2013).

14. Wike, E. L. *Data analysis: a statistical primer for psychology students*. (2017).

15. McDonald, S. Impairments in Social Cognition Following Severe Traumatic Brain Injury. *J. Int. Neuropsychol. Soc.* 19, 231–246 (2013).

16. Bland, J. M. & Altman, D. G. Statistics notes: Cronbach’s alpha. *BMJ* 314, 572–572 (1997).

17. Deyo, R. A. Measuring functional outcomes in therapeutic trials for chronic disease. *Control. Clin. Trials* 5, 223–240 (1984).

18. Deyo, R. A. & Centor, R. M. Assessing the responsiveness of functional scales to clinical change: an analogy to diagnostic test performance. *J. Chronic Dis.* 39, 897–906 (1986).

19. Liang, M. H. Longitudinal construct validity: establishment of clinical meaning in patient evaluative instruments. *Med. Care* 38, II84-90 (2000).

20. Wyrwich, K. W., Tierney, W. M. & Wolinsky, F. D. Further evidence supporting an SEM-based criterion for identifying meaningful intra-individual changes in health-related quality of life. *J. Clin. Epidemiol.* 52, 861–873 (1999).

21. Toussaint, A. *et al.* Sensitivity to change and minimal clinically important difference of the 7-item Generalized Anxiety Disorder Questionnaire (GAD-7). *J. Affect. Disord.* 265, 395–401 (2020).

22. Stratford, P. W. & Riddle, D. L. Assessing sensitivity to change: choosing the appropriate change coefficient. *Health Qual. Life Outcomes* 3, 23 (2005).

23. Cohen, J. A power primer. *Psychol. Bull.* 112, 155–159 (1992).

24. Weis, J. *et al.* Sensitivity to change of the EORTC quality of life module measuring cancer-related fatigue (EORTC QlQ-Fa12): Results from the international psychometric validation. *Psychooncology.* 28, 1753–1761 (2019).
