# Supplementary material for: Outcome prediction with a social cognitive battery: a multicenter longitudinal study
Source: NPJ Schizophr. 2021 May 26;7:30. doi: 10.1038/s41537-021-00160-5 (PMC8155046; doi:10.1038/s41537-021-00160-5)
Supplement: Supplementary file 2 — Reporting Summary [file 41537_2021_160_MOESM2_ESM.pdf]

nature research

Corresponding author(s): Eric Brunet-Gouet

Last updated by author(s): 2021/02/09

Reporting Summary

Nature Research wishes to improve the reproducibility of the work that we publish. This form provides structure for consistency and transparency in reporting. For further information on Nature Research policies, see our Editorial Policies and the Editorial Policy Checklist.

Statistics

For all statistical analyses, confirm that the following items are present in the figure legend, table legend, main text, or Methods section.

n/a	Confirmed	
☐	☒	The exact sample size (n) for each experimental group/condition, given as a discrete number and unit of measurement
☐	☒	A statement on whether measurements were taken from distinct samples or whether the same sample was measured repeatedly
☐	☒	The statistical test(s) used AND whether they are one- or two-sided<br>*Only common tests should be described solely by name; describe more complex techniques in the Methods section.*
☐	☒	A description of all covariates tested
☐	☒	A description of any assumptions or corrections, such as tests of normality and adjustment for multiple comparisons
☐	☒	A full description of the statistical parameters including central tendency (e.g. means) or other basic estimates (e.g. regression coefficient) AND variation (e.g. standard deviation) or associated estimates of uncertainty (e.g. confidence intervals)
☒	☐	For null hypothesis testing, the test statistic (e.g. F, t, r) with confidence intervals, effect sizes, degrees of freedom and P value noted<br>*Give P values as exact values whenever suitable.*
☒	☐	For Bayesian analysis, information on the choice of priors and Markov chain Monte Carlo settings
☒	☐	For hierarchical and complex designs, identification of the appropriate level for tests and full reporting of outcomes
☐	☒	Estimates of effect sizes (e.g. Cohen's d, Pearson's r), indicating how they were calculated

Our web collection on statistics for biologists contains articles on many of the points above.

Software and code

Policy information about availability of computer code

Data collection	SpeX
Data analysis	R

For manuscripts utilizing custom algorithms or software that are central to the research but not yet described in published literature, software must be made available to editors and reviewers. We strongly encourage code deposition in a community repository (e.g. GitHub). See the Nature Research guidelines for submitting code & software for further information.

Data

Policy information about availability of data

All manuscripts must include a data availability statement. This statement should provide the following information, where applicable:

- Accession codes, unique identifiers, or web links for publicly available datasets
- A list of figures that have associated raw data
- A description of any restrictions on data availability

The data presented here are not available for sharing. Any request should be addressed to EBG.

Field-specific reporting

Please select the one below that is the best fit for your research. If you are not sure, read the appropriate sections before making your selection.

☐ Life sciences ☒ Behavioural & social sciences ☐ Ecological, evolutionary & environmental sciences

For a reference copy of the document with all sections, see nature.com/documents/nr-reporting-summary-flat.pdf

Behavioural & social sciences study design

All studies must disclose on these points even when the disclosure is negative.

Study description	One year multicenter cohort study in centers of expertise for schizophrenia
Research sample	143 patients with schizophrenia (n = 103) or schizoaffective disorder (n = 40)
Sampling strategy	None
Data collection	PANSS, CGI, Evaco battery, Neuropsychological testing battery, Functioning, Quality of life
Timing	April 2013 and June 2017
Data exclusions	None
Non-participation	64 dropped out the study at the second evaluation
Randomization	None

Reporting for specific materials, systems and methods

We require information from authors about some types of materials, experimental systems and methods used in many studies. Here, indicate whether each material, system or method listed is relevant to your study. If you are not sure if a list item applies to your research, read the appropriate section before selecting a response.

Materials & experimental systems

n/a	Involved in the study	
☒	☐	Antibodies
☒	☐	Eukaryotic cell lines
☒	☐	Palaeontology and archaeology
☒	☐	Animals and other organisms
☐	☒	Human research participants
☐	☒	Clinical data
☒	☐	Dual use research of concern

Methods

n/a	Involved in the study	
☒	☐	ChIP-seq
☒	☐	Flow cytometry
☒	☐	MRI-based neuroimaging

Human research participants

Policy information about studies involving human research participants

Population characteristics	see above
Recruitment	Patients were recruited from seven Centers of Expertise for schizophrenia (Clermont-Ferrand, Créteil, Grenoble, Marseille, Montpellier, Strasbourg, and Versailles), making up a network established by the FondaMental Foundation. Patients were referred by their general practitioners or psychiatrists.
Ethics oversight	The local medical ethics committee (Comité de Protection des Personnes Ile-de-France XI) approved the study (2012-A00387-36).

Note that full information on the approval of the study protocol must also be provided in the manuscript.

Clinical data

Policy information about clinical studies

All manuscripts should comply with the ICMJE guidelines for publication of clinical research and a completed CONSORT checklist must be included with all submissions.

Clinical trial registration	NCT02901015
Study protocol	There is website access to the protocol.
Data collection	The data collection are holded by the INSERM/CESP/DEVPSY/DISAP research team.
Outcomes	Outcomes are evaluated with PSP (functioning) and SQoL (quality of life) tools. There is no primary outcome per se.
